# Similar major cardiovascular outcomes between pure statin and ezetimibe-statin in comparable intensity for type 2 diabetes with extremely atherosclerotic risks

**DOI:** 10.1038/s41598-021-86090-9

**Published:** 2021-03-23

**Authors:** Yu-Cheng Kao, Tien-Hsing Chen, Chi-Hung Liu, Jawl-Shan Hwang, Ching-Chung Hsiao, Yu-Sheng Lin, Chun-Tai Mao, Ming-Jui Hung, Yan-Rong Li

**Affiliations:** 1 Division of Cardiology, Department of Internal Medicine, Keelung Chang Gung Memorial Hospital, Keelung, Taiwan; 2 Biostatistical Consultation Center of Keelung Chang Gung Memorial Hospital, Keelung, Taiwan; 3 Stroke Center and Department of Neurology, Linkou Chang Gung Memorial Hospital, Taoyuan, Taiwan; 4 Division of Endocrinology and Metabolism, Department of Internal Medicine, Linkou Chang Gung Memorial Hospital, No. 5, Fu-Hsing St., Kueishan, Taoyuan Taiwan; 5 Kidney Research Center and Department of Nephrology, Taipei Chang Gung Memorial Hospital, Taipei, Taiwan; 6 Division of Cardiology, Department of Internal Medicine, Chiayi Chang Gung Memorial Hospital, Chiayi, Taiwan; 7 College of Medicine, Chang Gung University, Taoyuan, Taiwan

**Keywords:** Cardiology, Diseases, Endocrinology

## Abstract

Atorvastatin 40 mg (ATOR 40) and ezetimibe 10 mg/simvastatin 20 mg (EZ-SIM 20) have similar reductions of low-density lipoprotein cholesterol (LDL-C) but cardiovascular (CV) outcomes between these two therapies are unclear. Our real-world cohort study is to test the hypothesis of pleiotropic effects of purely higher dose statin on CV outcomes beyond similar reductions of LDL-C, especially for extremely CV risk patients. Between January 1, 2007 and December 31, 2013, a total of 3,372 patients with type 2 diabetes mellitus (T2DM) admitted due to acute coronary syndrome (ACS) or acute ischemic stroke (AIS) were selected as the study cohort from the Taiwan National Health Insurance Research Database. Clinical outcomes were evaluated by ATOR 40 group (n = 1686) matched with EZ-SIM 20 group (n = 1686). Primary composite outcome includes CV death, non-fatal myocardial infarction, and non-fatal stroke. Secondary composite outcome includes hospitalization for unstable angina (HUA), percutaneous coronary intervention (PCI), and coronary artery bypass grafting (CABG). With a mean follow-up of 2.4 years, no significant difference of primary composite outcome was observed between ATOR 40 and EZ-SIM 20 groups (subdistribution hazard ratio [SHR], 1.09; 95% confidence interval [CI], 0.95–1.25). Nevertheless, ATOR 40 group had lower risks of HUA (SHR, 0.50; 95% CI, 0.35–0.72), PCI (SHR, 0.82; 95% CI, 0.69–0.97) and CABG (SHR, 0.62; 95% CI, 0.40–0.97) than EZ-SIM 20 group. For T2DM patients after ACS or AIS, ATOR 40 and EZ-SIM 20 had similar major CV outcomes, which still supported the main driver for CV risk reductions is LDL-C lowering.

## Introduction

Type 2 diabetes mellitus (T2DM) is considered as a cardiovascular (CV) disease equivalent and is at risks of CV events and mortality which are two to four times than those in the general population^1,2^. In patients with T2DM, those with myocardial infarction or ischemic stroke are vulnerable for the further CV events^3,4^ and even classified as extremely atherosclerotic cardiovascular disease (ASCVD) risk patients by a recent definition^5^. For these patients, high-intensity statins should be administered to achieve low-density lipoprotein cholesterol (LDL-C) ≥ 50% reduction or to meet the individual goal of LDL-C according to the different guidelines^5–8^. For either primary or secondary prevention of CV diseases, the concept of “the lower, the better” is verified from the results of numerous trials 3-hydroxy-3-methylglutaryl coenzyme A (HMG-CoA) reductase inhibitors (statins) and landmark studies of non-statin LDL-C lowering agents, such as ezetimibe^9,10^ or proprotein convertase subtilsin-kexin type 9 (PCSK9) inhibitors^11,12^. Many studies and the result of meta-analysis from Cholesterol Treatment Trialists' (CTT) have documented that LDL-C is the driving force to lower CV risks^13–15^. However, one clinical question could be interesting and that is “Is there a different CV outcome between pure statin and ezetimibe-statin therapies when having a comparable reduction of LDL-C or goal ?”.

Atorvastatin 40 mg (ATOR 40) and ezetimibe 10 mg/simvastatin 20 mg (EZ-SIM 20) theoretically have a similar and comparable LDL-C lowering effect^16,17^ and could be categorized as a high-intensity LDL-C lowering agent^18,19^. Nevertheless, ATOR 40 may have more reduction of high sensitivity C-reactive protein (hs-CRP) compared to EZ-SIM 20 (reduction of hs-CRP: 28.6% vs. 21.4%)^17^ but another study showed similar CRP reductions between EZ-SIM and ATOR at averaged across doses and at each milligram-equivalent statin dose in comparison^20^. Besides, the pleiotropic effects of statins, such as decreasing oxidative stress, improving endothelial dysfunction, lowering endothelial cell apoptosis, alleviating inflammation and beneficial to the immune system which are independent of LDL-C reduction and regardless of the level of LDL-C have been supported by the literatures^21–23^. Some researches which were conducted with randomizing healthy volunteers or patients with coronary artery disease by comparing higher dose statins with a combination of lower dose statins and ezetimibe revealed more improvements in endothelial function and vascular inflammation by the higher dose statins in a comparable reduction of LDL-C^24–27^ but some other studies did not observe the same findings^28–30^. Therefore, even in a similar reduction of LDL-C, whether purely higher dose statins are better than a combination of ezetimibe and lower dose statins is still inconclusive. Moreover, these studies are not designed specifically to examine the major and solid CV outcomes, such as CV death, non-fatal myocardial infarction (MI), non-fatal stroke, hospitalization for unstable angina (HUA), percutaneous coronary intervention (PCI), and coronary artery bypass grafting (CABG). Therefore, although there is a similar LDL-C lowering effect from ATOR 40 and EZ-SIM 20, the CV outcomes between the purely higher dose statin and ezetimibe-statin therapies are still unclear, especially for extremely CV risk patients who tend to be more vulnerable to suffer from further major CV diseases within a relatively short period and could significantly get more CV benefits with adequate treatments because the effects of statins are better in secondary prevention than those in primary prevention. Given the uncertain CV effects between ATOR 40 and EZ-SIM 20 on patients with extremely CV risks, we used data from Taiwan’s National Health Insurance Research Database (NHIRD) to conduct a nationwide and population-based cohort study to test the hypothesis of pleiotropic effects of the purely higher dose statins playing a role in CV outcomes beyond similar reductions of LDL-C by analyzing T2DM patients after acute coronary syndrome (ACS) or acute ischemic stroke (AIS) who were treated ATOR 40 and EZ-SIM 20, respectively.

## Materials and methods

### Data source

The National Health Insurance (NHI) program in Taiwan is since 1995 and covers the medical needs of 99.8% of 23 million people in the country. All standardized information and data of in this healthcare services are prospectively recorded by the NHIRD and contains inpatient and outpatient data, including date of birth, sex, diagnosis codes (International Classification of Diseases, Ninth Revision, Clinical Modification [ICD-9-CM] codes), drug prescriptions, surgical procedures, admission dates, hospitalizations, discharge dates, and expenditure which has been validated by the previous researches^31–35^. The ethics approval and protocol of this study were approved by the Ethics Institutional Review Board of Linkou Chang Gung Memorial Hospital.

### Identification of study cohort

**Figure 1:**
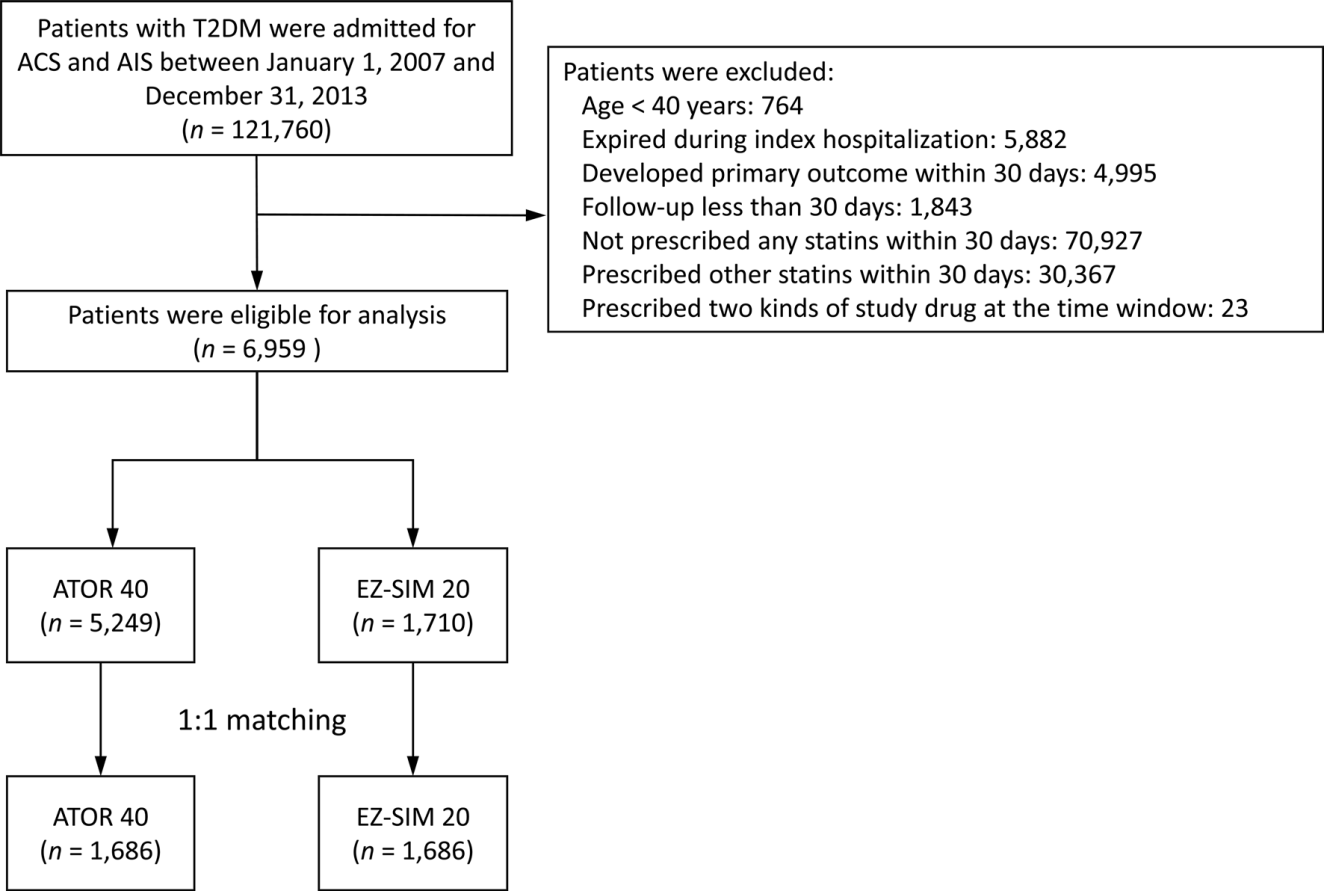
Flow chart of selections of the study cohort.

The current study was a nationwide population-based, observational and open cohort using retrospective-collected data from the NHIRD between January 1, 2007 and December 31, 2013. We identified patients with diagnoses of T2DM (excluding type 1 diabetes mellitus) between January 1, 2007 and December 31, 2013. Only 121,760 T2DM patients admitted with a principal diagnosis of ACS or AIS were included for analysis. After relevant exclusion, 6,959 patients with T2DM aged ≥ 40 years who were admitted due to ACS or AIS were eligible for analyses and after propensity score matching (PSM) in a 1:1 ratio, a total of 3,372 subjects (1,686 subjects in the ATOR 40 group and 1,686 subjects in the EZ-SIM 20 group) were included into the final analysis (Fig. 1). The definition of the index date was the date of discharge. The follow-up period was based on the index date to the date of death or December 31, 2013.

### Exposure of study statin

During the study period, patients received these two kinds of statin-containing drugs according to the lipid-lowering guidelines in Taiwan. T2DM patients with elevated level of LDL-C ≥ 130 mg/dL received NHI-paid LDL-C lowering agents to reach the therapeutic goal of LDL-C. The treatment goal of T2DM patients with established CV diseases by NHI in Taiwan was less than 100 mg/dL during the study period. Eligible patients who received study drugs within 30 days of the index date were divided into two groups according to the prescribed LDL-C lowering agents, the ATOR 40 group and the EZ-SIM 20 group, respectively. This definition of exposure of study drugs was reported in our previous publications^36–38^.

### Ascertainment of T2DM, ACS and AIS

The diagnosis of T2DM is validated according to ICD-9-CM codes where at least four visits of outpatient corresponded to an accuracy of 95.7% and with prescriptions of oral anti-diabetic agents corresponded to an accuracy of 99%^39^. We identified patients with T2DM based on diagnosis code and anti-diabetic agents simultaneously. The inclusions of ACS and AIS were requiring a principal diagnosis of admission. The diagnosis codes of ACS and AIS have been validated in previous NHIRD studies that have obtained high positive predictive values with ≥ 95%^31,33,34^.

### Covariates measurements

Comorbidities and histories of event at baseline were identified based on ICD-9-CM diagnosis codes (Supplemental Table S1). Comorbidities were defined as at least two outpatient visits or anyone inpatient diagnosis in the previous year of the index date. Histories of event was detected using anyone inpatient diagnosis before the index date which can be tracked to year 1997. The baseline medication was defined as the medications prescribed in the outpatient visits or the refill in the pharmacy within 30 days of the index date. For evaluation of adherence, we analyzed the rate of refilling medication, which was medication possession ratio (MPR). After matching, there was no difference between ATOR 40 and EZ-SIM 20 (Supplemental Table S2).

### Ascertainment of primary, secondary and safety outcomes

The primary composite outcome was an endpoint of CV death, non-fatal MI, and non-fatal stroke. The occurrences of ACS and AIS were defined as a principal inpatient diagnosis. The definition of CV death is the criteria of the Standardized Definitions for Cardiovascular and Stroke Endpoint Events in Clinical Trials by the FDA in the United States.

The secondary composite CV outcome was defined as hospitalization for unstable angina (HUA), percutaneous coronary intervention (PCI), and coronary artery bypass grafting (CABG). The occurrence of HUA was defined as a principal inpatient diagnosis. Information of PCI and CABG was extracted using the Taiwan NHI reimbursement codes of inpatient claims. Other secondary outcomes included hospitalization for heart failure (HHF), and all-cause mortality^40^.

The safety outcomes were defined as hemorrhagic stroke, acute hepatitis, rhabdomyolysis, newly diagnosed dementia and newly diagnosed cancer during the period of follow-up.

### Statistical analysis

To decrease bias due to confounding when comparing treatment effects between the ATOR 40 and EZ-SIM 20 groups, we conducted a propensity score matching analysis. The propensity score was the predicted probability of being the in one group given the values of covariates in the logistic regression. The selected covariates to calculate propensity score were listed in Table 1 where the follow up year was replaced with the index date. We adopted a greedy nearest neighbor algorithm with a caliper of 0.2 times the standard deviation of the logit of propensity score, with random matching order and without replacement. Matching quality was assessed using the absolute value of the standardized difference (STD) between the groups after matching, where a value lower than 0.1 represented negligible difference between the groups.

**Table 1:** Characteristics of the study patients before and after propensity score matching.

Characteristics	Data before matching			Data after matching		
	ATOR 40 (*n* = 5249)	EZ-SIM 20 (*n *= 1710)	STD	ATOR 40 (*n* = 1686)	EZ-SIM 20 (*n *= 1686)	STD
**Etiology**						
ACS	2118 (40.4)	594 (34.7)	0.12	591 (35.1)	591 (35.1)	<0.01
AIS	3131 (59.6)	1116 (65.3)	−0.12	1095 (64.9)	1095 (64.9)	<0.01
Age at index date, years	66.4 ± 10.6	66.1 ± 10.5	0.03	66.3 ± 10.7	66.1 ± 10.5	0.02
**Age group**						
40–64 years	2368 (45.1)	788 (46.1)	−0.02	748 (44.4)	775 (46.0)	−0.03
65–74 years	1668 (31.8)	540 (31.6)	<0.01	543 (32.2)	533 (31.6)	0.01
75–84 years	1049 (20.0)	341 (19.9)	<0.01	350 (20.8)	337 (20.0)	0.02
≥ 85 years	164 (3.1)	41 (2.4)	0.04	45 (2.7)	41 (2.4)	0.02
**Gender**						
Male	2932 (55.9)	892 (52.2)	0.07	861 (51.1)	885 (52.5)	−0.03
Female	2317 (44.1)	818 (47.8)	−0.07	825 (48.9)	801 (47.5)	0.03
DM duration, years	9.7 ± 3.5	10.5 ± 3.6	−0.24	10.4 ± 3.5	10.5 ± 3.6	−0.01
**DM duration group**						
0–5 years	843 (16.1)	206 (12.0)	0.12	198 (11.7)	204 (12.1)	−0.01
6–10 years	2382 (45.4)	603 (35.3)	0.21	621 (36.8)	602 (35.7)	0.02
11–15 years	1977 (37.7)	863 (50.5)	−0.26	835 (49.5)	846 (50.2)	−0.01
≥ 16 years	47 (0.9)	38 (2.2)	−0.11	32 (1.9)	34 (2.0)	−0.01
No. of outpatient DM visit in previous year	13.6 ± 8.8	14.1 ± 9.4	−0.05	13.8 ± 8.7	14.0 ± 9.3	−0.03
HbA1c exam in the previous year	2.4 ± 1.9	2.7 ± 2.0	−0.17	2.6 ± 2.0	2.7 ± 2.0	−0.04
**History of event**						
Previous ischemic stroke	956 (18.2)	333 (19.5)	−0.03	328 (19.5)	328 (19.5)	<0.01
Old myocardial infarction	372 (7.1)	141 (8.2)	−0.04	133 (7.9)	136 (8.1)	−0.01
Heart failure	594 (11.3)	201 (11.8)	−0.01	210 (12.5)	197 (11.7)	0.02
VTE: PE or DV	37 (0.7)	21 (1.2)	−0.05	18 (1.1)	19 (1.1)	−0.01
**Comorbidity**						
Chronic kidney disease (CKD)						
None	3735 (71.2)	1126 (65.8)	0.11	1126 (66.8)	1117 (66.3)	0.01
Non-dialysis CKD	1277 (24.3)	512 (29.9)	−0.13	489 (29.0)	498 (29.5)	−0.01
Dialysis	237 (4.5)	72 (4.2)	0.01	71 (4.2)	71 (4.2)	<0.01
Gout	471 (9.0)	161 (9.4)	−0.02	142 (8.4)	158 (9.4)	−0.03
Atrial fibrillation	306 (5.8)	98 (5.7)	<0.01	98 (5.8)	98 (5.8)	<0.01
Peripheral arterial disease	267 (5.1)	101 (5.9)	−0.04	90 (5.3)	99 (5.9)	−0.02
Hypertension	4497 (85.7)	1503 (87.9)	−0.07	1461 (86.7)	1480 (87.8)	−0.03
Dyslipidemia	3649 (69.5)	1274 (74.5)	−0.11	1241 (73.6)	1254 (74.4)	−0.02
Chronic obstructive pulmonary disease	344 (6.6)	114 (6.7)	<0.01	110 (6.5)	112 (6.6)	<0.01
Malignancy	255 (4.9)	97 (5.7)	−0.04	93 (5.5)	96 (5.7)	−0.01
Cirrhosis	45 (0.9)	9 (0.5)	0.04	5 (0.3)	9 (0.5)	−0.04
HBV infection	68 (1.3)	17 (1.0)	0.03	16 (0.9)	17 (1.0)	−0.01
HCV infection	61 (1.2)	21 (1.2)	−0.01	24 (1.4)	21 (1.2)	0.02
Alcoholism	33 (0.6)	8 (0.5)	0.02	6 (0.4)	8 (0.5)	−0.02
Autoimmune disease	71 (1.4)	30 (1.8)	−0.03	31 (1.8)	29 (1.7)	0.01
Previous coronary intervention	690 (13.1)	297 (17.4)	−0.12	270 (16.0)	289 (17.1)	−0.03
**Non-DM medication**						
Aspirin	4803 (91.5)	1526 (89.2)	0.08	1532 (90.9)	1506 (89.3)	0.05
Clopidogrel	2608 (49.7)	755 (44.2)	0.11	739 (43.8)	746 (44.2)	−0.01
Anticoagulant (Warfarin or NOAC)	293 (5.6)	82 (4.8)	0.04	75 (4.4)	82 (4.9)	−0.02
ACEI/ARB	3768 (71.8)	1238 (72.4)	−0.01	1230 (73.0)	1220 (72.4)	0.01
β-blocker	2559 (48.8)	795 (46.5)	0.05	776 (46.0)	785 (46.6)	−0.01
CCB	2718 (51.8)	887 (51.9)	<0.01	885 (52.5)	875 (51.9)	0.01
Digoxin	212 (4.0)	62 (3.6)	0.02	75 (4.4)	62 (3.7)	0.04
NSAID/COX-2 inhibitor	1272 (24.2)	461 (27.0)	−0.06	442 (26.2)	452 (26.8)	−0.01
Diuretic	1149 (21.9)	384 (22.5)	−0.01	390 (23.1)	377 (22.4)	0.02
Spironolactone	452 (8.6)	115 (6.7)	0.07	125 (7.4)	115 (6.8)	0.02
Fibrate	225 (4.3)	159 (9.3)	−0.20	138 (8.2)	144 (8.5)	−0.01
**Hypoglycemic drugs**						
Biguanide	2873 (54.7)	851 (49.8)	0.10	863 (51.2)	843 (50.0)	0.02
Sulfonylurea	2959 (56.4)	833 (48.7)	0.15	849 (50.4)	827 (49.1)	0.03
Thiazolidinedione	453 (8.6)	201 (11.8)	−0.10	192 (11.4)	194 (11.5)	<0.01
Alpha-glucosidase	954 (18.2)	289 (16.9)	0.03	275 (16.3)	287 (17.0)	−0.02
Non-SU insulin secretagogue (Glinide)	795 (15.1)	265 (15.5)	−0.01	265 (15.7)	262 (15.5)	<0.01
Insulin	2893 (55.1)	886 (51.8)	0.07	862 (51.1)	873 (51.8)	−0.01
DPP-4 inhibitor	897 (17.1)	512 (29.9)	−0.31	499 (29.6)	495 (29.4)	0.01

ATOR 40, atorvastatin 40mg; EZ-SIM 20, ezetimibe 10mg/simvastatin 20mg; STD, standardized difference; ACS, acute coronary syndrome; AIS, acute ischemic stroke; DM, diabetes mellitus; VTE, venous thromboembolism; PE, pulmonary embolism; DVT, deep vein thrombosis; HBV, hepatitis B virus; HCV, hepatitis C virus; NOAC, novel oral anticoagulant; ACEI, angiotensin-converting-enzyme inhibitor; ARB, angiotensin receptor blocker; CCB, calcium channel blocker; NSAID, non-steroidal anti-inflammatory drug; COX-2, cyclo-oxygenase-2; DPP-4, dipeptidyl peptidase-4.

Data were presented as frequency and percentage or mean ± standard deviation.

We compared the risks of all-cause mortality between the groups by using the Cox proportional hazards model. The risks of other time-to-event outcomes in the two groups were compared using the Fine and Gray subdistribution hazard model that considered death a competing risk. The within-pair clustering of outcomes after propensity score matching was accounted for by using a robust standard error (the marginal model)^41^. The unadjusted cumulative incidence function of the outcomes was calculated and plotted under a subdistribution hazard model. The study group (ATOR 40 vs. EZ-SIM 20) was the only explanatory variable in survival analyses.

A *post-hoc* subgroup analysis was performed to determine whether the subdistribution hazard ratios of secondary composite CV outcomes for the ATOR 40 and EZ-SIM 20 groups were similar in the subgroups. A two-sided *P* value of less than 0.05 was considered statistically significant and no adjustment for multiple testing (multiplicity) was made in this study. All statistical analyses were performed using SAS version 9.4 (SAS Institute, Cary, NC, USA), including the procedures of ‘*psmatch’* for PSM, ‘*phreg’* for survival analyses, and the macro of ‘*%cif’* for the cumulative incidence function.

### Ethics approval and consent to participate

The protocol of this study and informed consent of all participants were approved by the Ethics Institutional Review Board of Chang Gung Memorial Hospital.

### Consent for publication

Authors give full consents for publication of this present article.

## Results

### Demographics and clinical characteristics

**Figure 2:**
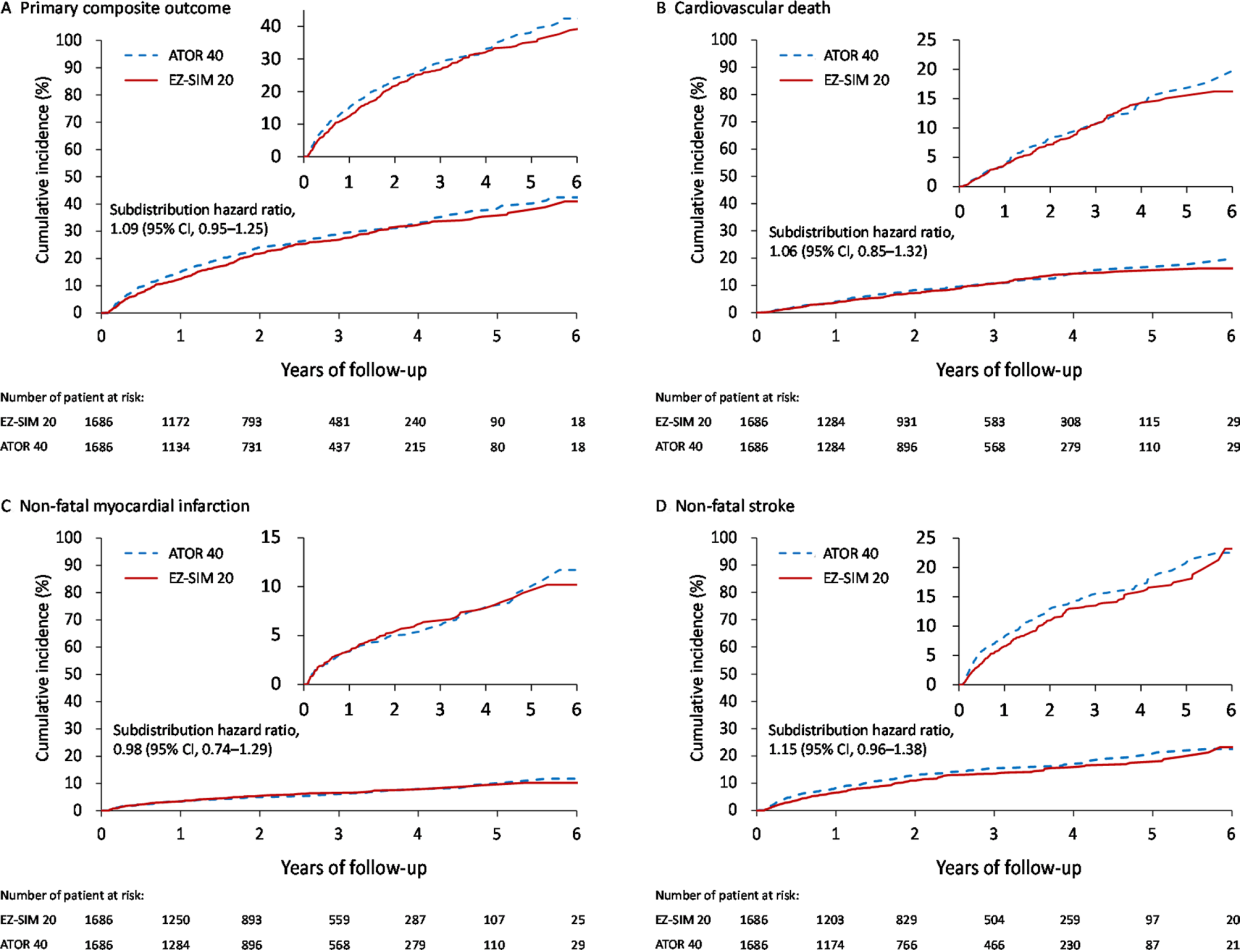
The cumulative incidence of the primary composite outcome (**A**), and the individual outcomes of cardiovascular death (**B**), non-fatal myocardial infarction (**C**) and non-fatal stroke (**D**) in the propensity score matched cohorts.

In total, 6,959 patients with T2DM who were admitted for ACS or AIS between January 1, 2007 and December 31, 2013, were eligible for this study. Of those, 5249 (74.4%) patients were prescribed with ATOR 40 and 1710 (24.6%) ones were prescribed with EZ-SIM 20. After application of PSM with 1:1 ratio, 1686 patients in either groups (Fig. 2). After PSM, all values of absolute STD were lower than 0.1, which stands for negligible differences in demographics, comorbidities, and medications at baseline between these two groups (right panel of Table 1).

The mean follow-up period was 2.4 years (standard deviation [SD], 1.6 years) and the maximum follow-up duration was 6.9 years. The mean age of the patients at baseline was 66.2 years (SD, 10.6 years). The most common co-morbidity was hypertension (86.7% vs. 87.8%), followed by dyslipidemia (73.6% vs. 74.4%) and chronic kidney disease (33.2% vs. 33.7%) in the ATOR 40 group and the EZ-SIM 20 group, respectively. In addition, patients with old MI and old ischemic stroke in the ATOR 40 group were 7.9 and 19.5%, respectively; in the EZ-SIM 20 group, those with old MI and old ischemic stroke were 8.1 and 19.5%, respectively (Table 1).

### Primary composite outcome

The primary composite outcome occurred in 422 patients (25.0%) in the ATOR 40 group and 398 patients (23.6%) in the EZ-SIM 20 group (subdistribution hazard ratio [SHR], 1.09; 95% confidence interval [CI], 0.95–1.25; Table 2; Fig. 2A). Regarding the individual composite outcomes, non-significant difference of risks for CV death (SHR, 1.06; 95% CI, 0.85–1.32), non-fatal MI (SHR, 0.98; 95% CI, 0.74–1.29), and non-fatal stroke (SHR, 1.15; 95% CI, 0.96–1.38) were observed between these two groups (Table 2; Fig. 2B-2D).

**Table 2:** Clinical outcomes between the study cohorts.

Variable	ATOR 40 (*n* = 1686)	EZ-SIM 20 (*n *= 1686)	HR or SHR (95% CI)	*P* value
Primary composite outcome	422 (25.0)	398 (23.6)	1.09 (0.95–1.25)	0.207
CV death	160 (9.5)	152 (9.0)	1.06 (0.85–1.32)	0.596
Non-fatal myocardial infarction	97 (5.8)	100 (5.9)	0.98 (0.74–1.29)	0.861
Non-fatal stroke	225 (13.3)	200 (11.9)	1.15 (0.96–1.38)	0.134
Secondary composite outcome	255 (15.1)	326 (19.3)	0.77 (0.66–0.89)	<0.001
Hospitalization for unstable angina	42 (2.5)	82 (4.9)	0.50 (0.35–0.72)	<0.001
PCI	215 (12.8)	261 (15.5)	0.82 (0.69–0.97)	0.020
CABG	28 (1.7)	45 (2.7)	0.62 (0.40–0.97)	0.038
**Other secondary outcomes**				
All-cause mortality	292 (17.3)	273 (16.2)	1.09 (0.92–1.28)	0.324
Hospitalization for heart failure	145 (8.6)	138 (8.2)	1.05 (0.84–1.32)	0.676
**Safety outcomes**				
Hemorrhagic stroke	15 (0.9)	21 (1.2)	0.72 (0.38–1.38)	0.323
Acute hepatitis	10 (0.6)	4 (0.2)	2.52 (0.79–8.02)	0.118
Rhabdomyolysis	5 (0.3)	8 (0.5)	0.63 (0.21–1.91)	0.411
New diagnosed dementia	85 (5.0)	87 (5.2)	0.98 (0.73–1.33)	0.910
New diagnosed malignancy	73 (4.3)	65 (3.9)	1.14 (0.82–1.57)	0.437

ATOR 40, atorvastatin 40mg; EZ-SIM 20, ezetimibe 10mg/simvastatin 20mg; HR, hazard ratio; SHR, subdistribution hazard ratio; CI, confidence interval; CV, cardiovascular; PCI, percutaneous coronary intervention; CABG, coronary artery bypass grafting.

Data were presented as frequency and percentage.

### Secondary composite outcome and other clinical outcomes

The secondary composite outcome occurred in 255 patients (15.1%) in the ATOR 40 group and 326 patients (19.3%) in the EZ-SIM 20 group (SHR, 0.77; 95% CI, 0.66–0.89; Table 2; Supplemental Figure S1A). Patients in the ATOR 40 group had lower risks of HUA (SHR, 0.50; 95% CI, 0.35–0.72), PCI (SHR, 0.82; 95% CI, 0.69–0.97) and CABG (SHR, 0.62; 95% CI, 0.40–0.97) than those in the EZ-SIM 20 group (Table 2; Supplemental Figure S1B-D). The risks of HHF (SHR, 1.05; 95% CI, 0.84–1.32) and all-cause mortality (hazard ratio, 1.09; 95% CI, 0.92–1.28) were similar between the ATOR 40 group and the EZ-SIM 20 group (Table 2).

### Safety outcomes

The ATOR 40 and EZ-SIM 20 groups did not differ significantly in terms of incidence of hemorrhagic stroke (0.9% vs. 1.2%; P = 0.323), acute hepatitis (0.6% vs. 0.2%; P = 0.118), rhabdomyolysis (0.3% vs. 0.5%; P = 0.411), newly diagnosed dementia (5.0% vs. 5.2%; P = 0.910), or newly diagnosed malignancy (4.3% vs. 3.9%; P = 0.437) (Table 2).

### Subgroup analysis

There was a significant result of the secondary composite outcome between these two groups. Therefore, a *post-hoc* subgroup analysis was performed to evaluate whether the SHRs of the secondary composite outcome were similar in the selected subgroups. The results demonstrated that the effect of ATOR 40 did not differ significantly in the subgroups of age, gender, duration of T2DM, admitted due to ACS or AIS, heart failure, chronic kidney disease, atrial fibrillation, hypertension, and dyslipidemia (Supplemental Figure S2). Noticeably, the values of all SHRs were less than 1 which favored the ATOR 40 group regardless of the statistical significance.

## Discussion

In this nationwide, population-based, non-crossover and observational cohort study, we tested the hypothesis of “the pleiotropic effects of purely higher dose statin may play a role in CV benefits than a combination of ezetimibe and lower dose statin in a comparable reduction of LDL-C”. The primary composite outcome (CV death, non-fatal MI and non-fatal stroke) was no different between the ATOR 40 group and the EZ-SIM 20 group in T2DM patients after ACS or AIS. Therefore, our results of study supported the concept that LDL-C is a key role in the pathogenesis of ASCVD^42,43^ and the capacity of medication to lower of LDL-C regardless of statin or non-statin therapies (ezetimibe and PCSK9 inhibitors) is definitely the first concern rather than the pleiotropic effects. Treatment with ATOR 40 had the lower risks of HUA, PCI and CABG than EZ-SIM 20 which is compatible with coronary plaque stabilization is independent of LDL-C reduction^44^, however, secondary endpoints are not robust enough to draw a causal effect and should be interpreted carefully.

As studies aforementioned in the section of introduction, whether statin's clinical CV benefits are partially due to its pleiotropic effects or just due to the reduction of LDL-C and even in a equal intensity of LDL-C lowering, whether purely higher dose statin is better than a combination of ezetimibe and lower dose statin are still debated; positive findings were limited to the discovery of improvements of endothelial function and vascular inflammation which could only be a surrogate marker for CV outcomes in our real-world patients. Therefore, the valuable strength of our research is that it is the first real-world and nationwide population-based cohort study to evaluate the solid CV outcomes of the pleiotropic effects of statins at the similar reduction of LDL-C and still supported the main driver for CV risk reductions is LDL-C lowering. The similar or comparable reduction of LDL-C brings the similarly major CV outcomes.

However, according to our data, something in detail should be more addressed as follows. Although the reduction of LDL-C of ATOR 40 is similar to EZ-SIM 20, the reduction of LDL-C by ATOR 40 is still less than that by EZ-SIM 20 numerically according to the previous data; the average reductions of LDL-C by ATOR 40 is 48.3–49%^17,45–47^ but those by EZ-SIM 20 is 50.6–51.9%^17,48,49^. Therefore, for primary composite outcome, 398 patients (23.6%) occurred in the EZ-SIM 20 group and 422 patients (25.0%) occurred in the ATOR 40 group. The event numbers in the EZ-SIM 20 group was not statistically significant lower than those in the ATOR 40 group which still supported the main driver for CV risk reduction is LDL-C lowering. IMPROVE-IT study showed that the CV risk reduction with additional ezetimibe was precise as the same as that predicted by the CTT analysis, which suggests that reduction of LDL-C per se stands for reducing the risk of CV disease and supports the LDL hypothesis, rather than pleiotropic effects^50^.

### Limitation

Our present study has some major limitations. First, laboratory parameters including levels of glycated hemoglobin, lipid profile, hs-CRP, blood pressure and body mass index were not available in the registry data from NHIRD in Taiwan. Therefore, we included a wide range of baseline comorbidities and medications with propensity score matching to minimize potential selection bias such as confounding by indication and to make our two study groups well-balanced when comparing treatment effects. Because the lab data in the NHIRD database were not available, the actual effects of lipid-lowering agents were unknown and the patients could only be assumed to have theoretically therapeutic effects based on previous literatures. The record of smoking was lacking in the NHIRD database and smoking is an important confounding factor of CV outcomes. Therefore, chronic obstructive pulmonary disease (COPD) was used as a proxy variable instead of smoking for matching at baseline to mitigate this important confounding factor because smoking is strongly associated with the prevalence of COPD^51^. Second, according to the previous NHI regulations in Taiwan for data request, all data we have is to the date on December 31, 2013. Therefore, the 2.4-year mean follow-up is short for this type of study. Third, unfortunately in this claim database study, we had difficulty in clarifying the subtypes of the upcoming ischemic stroke. Because the strong association between cholesterol and carotid atherosclerosis supporting that cholesterol is a main pathogenesis of the large artery ischemic stroke, the large artery ischemic stroke is one subtype of ischemic strokes that could be proven to get benefits from statins treatment. Finally, the present retrospectively observational study is unable to draw a causal effect due to the possibility of unmeasured confounding; therefore, the future randomized controlled trials can be needed to confirm our findings.

In spite of these limitations, our real-world and nationwide population-based cohort study is still valuable to answer the uncertain question and to fill the gap of evidence of whether pleiotropic effects by pure statins play a role on CV outcomes beyond a similar reduction of LDL-C.

## Conclusions

In summary, our results of study supported the concept that LDL-C is a key role in the pathogenesis of ASCVD and the capacity of medication to lower of LDL-C regardless of statin or non-statin therapies (ezetimibe and PCSK9 inhibitors) is definitely the first concern rather than the pleiotropic effects.

## Supplementary Information

Supplementary Information 1.

Supplementary Information 2.

Supplementary Information 3.

Supplementary Information 4.

Supplementary Legends.

## Data Availability

This datasets used and analyzed in this study are available from the corresponding author on reasonable requests.

## References

[1] Haffner SM, Lehto S, Ronnemaa T, Pyorala K, Laakso M (1998). Mortality from coronary heart disease in subjects with type 2 diabetes and in nondiabetic subjects with and without prior myocardial infarction. N. Engl. J. Med..

[2] Rawshani A, Rawshani A, Franzen S (2018). Risk factors, mortality, and cardiovascular outcomes in patients with type 2 diabetes. N. Engl. J. Med..

[3] Boccara F, Cohen A (2004). Interplay of diabetes and coronary heart disease on cardiovascular mortality. Heart.

[4] Shou J, Zhou L, Zhu S, Zhang X (2015). Diabetes is an independent risk factor for stroke recurrence in stroke patients: a meta-analysis. J. Stroke Cerebrovasc. Dis..

[5] Garber AJ, Abrahamson MJ, Barzilay JI (2019). Consensus statement by the American Association of Clinical Endocrinologists and American College of Endocrinology on the Comprehensive Type 2 Diabetes Management Algorithm-2019 Executive Summary. Endocr. Pract..

[6] Li YH, Ueng KC, Jeng JS (2017). 2017 Taiwan lipid guidelines for high risk patients. J. Formos. Med. Assoc..

[7] Grundy, S.M., Stone, N.J., Bailey, A.L., et al., AHA/ACC/AACVPR/AAPA/ABC/ACPM/ADA/AGS/APhA/ASPC/NLA/PCNA Guideline on the Management of Blood Cholesterol: A Report of the American College of Cardiology/American Heart Association Task Force on Clinical Practice Guidelines. J Am Coll Cardiol. 2018.

[8] Disease C, Management R (2019). Standards of medical care in diabetes-2019. Diabetes Care.

[9] Cannon CP, Blazing MA, Giugliano RP (2015). Ezetimibe added to statin therapy after acute coronary syndromes. N. Engl. J. Med..

[10] Ouchi Y, Sasaki J, Arai H (2019). Ezetimibe lipid-lowering trial on prevention of atherosclerotic cardiovascular disease in 75 or older (EWTOPIA 75): a randomized, controlled trial. Circulation.

[11] Schwartz GG, Steg PG, Szarek M (2018). Alirocumab and cardiovascular outcomes after acute coronary syndrome. N. Engl. J. Med..

[12] Sabatine MS, Giugliano RP, Keech AC (2017). Evolocumab and clinical outcomes in patients with cardiovascular disease. N. Engl. J. Med..

[13] Stamler J, Wentworth D, Neaton JD (1986). Is relationship between serum cholesterol and risk of premature death from coronary heart disease continuous and graded? Findings in 356,222 primary screenees of the Multiple Risk Factor Intervention Trial (MRFIT). JAMA.

[14] Ballantyne CM, Grundy SM, Oberman A (2000). Hyperlipidemia: diagnostic and therapeutic perspectives. J. Clin. Endocrinol. Metab..

[15] Baigent C, Blackwell L (2010). Cholesterol Treatment Trialists’ (CTT) Collaboration. Efficacy and safety of more intensive lowering of LDL cholesterol: a meta-analysis of data from 170,000 participants in 26 randomised trials. Lancet.

[16] Stone NJ, Robinson JG, Lichtenstein AH (2014). 2013 ACC/AHA guideline on the treatment of blood cholesterol to reduce atherosclerotic cardiovascular risk in adults: a report of the American College of Cardiology/American Heart Association Task Force on Practice Guidelines. Circulation.

[17] Ballantyne CM, Abate N, Yuan Z, King TR, Palmisano J (2005). Dose-comparison study of the combination of ezetimibe and simvastatin (Vytorin) versus atorvastatin in patients with hypercholesterolemia: the Vytorin Versus Atorvastatin (VYVA) study. Am Heart J..

[18] Galin ID, Smith DA (2006). Dose-comparison study of the combination of ezetimibe and simvastatin (Vytorin) versus atorvastatin in patients with hypercholesterolemia: the Vytorin Versus Atorvastatin (VYVA) Study. Am Heart J..

[19] Masana L, Pedro-Botet J, Civeira F (2015). IMPROVE-IT clinical implications. Should the "high-intensity cholesterol-lowering therapy" strategy replace the "high-intensity statin therapy"?. Atherosclerosis.

[20] Pearson T, Ballantyne C, Sisk C (2007). Comparison of effects of ezetimibe/simvastatin versus simvastatin versus atorvastatin in reducing C-reactive protein and low-density lipoprotein cholesterol levels. Am J Cardiol..

[21] Liao JK, Laufs U (2005). Pleiotropic effects of statins. Annu. Rev. Pharmacol. Toxicol..

[22] Oesterle A, Laufs U, Liao JK (2017). Pleiotropic effects of statins on the cardiovascular system. Circ. Res..

[23] Oesterle A, Liao JK (2019). The pleiotropic effects of statins—from coronary artery disease and stroke to atrial fibrillation and ventricular tachyarrhythmia. Curr. Vasc. Pharmacol..

[24] Liu PY, Liu YW, Lin LJ, Chen JH, Liao JK (2009). Evidence for statin pleiotropy in humans: differential effects of statins and ezetimibe on rho-associated coiled-coil containing protein kinase activity, endothelial function, and inflammation. Circulation.

[25] Fichtlscherer S, Schmidt-Lucke C, Bojunga S (2006). Differential effects of short-term lipid lowering with ezetimibe and statins on endothelial function in patients with CAD: clinical evidence for 'pleiotropic' functions of statin therapy. Eur. Heart J..

[26] Rudofsky G, Reismann P, Groener JB (2012). Identical LDL-cholesterol lowering but non-identical effects on NF-kappaB activity: high dose simvastatin vs combination therapy with ezetimibe. Atherosclerosis.

[27] Matsue Y, Matsumura A, Suzuki M, Hashimoto Y, Yoshida M (2013). Differences in action of atorvastatin and ezetimibe in lowering low-density lipoprotein cholesterol and effect on endothelial function: randomized controlled trial. Circ. J..

[28] Kawagoe Y, Hattori Y, Nakano A (2011). Comparative study between high-dose fluvastatin and low-dose fluvastatin and ezetimibe with regard to the effect on endothelial function in diabetic patients. Endocr. J..

[29] Pesaro AE, Serrano CV, Fernandes JL (2012). Pleiotropic effects of ezetimibe/simvastatin vs. high dose simvastatin. Int. J. Cardiol..

[30] Westerink J, Deanfield JE, Imholz BP (2013). High-dose statin monotherapy versus low-dose statin/ezetimibe combination on fasting and postprandial lipids and endothelial function in obese patients with the metabolic syndrome: The PANACEA study. Atherosclerosis.

[31] Hsieh CY, Chen CH, Li CY, Lai ML (2015). Validating the diagnosis of acute ischemic stroke in a National Health Insurance claims database. J. Formos Med. Assoc..

[32] Chang CH, Lee YC, Tsai CT (2014). Continuation of statin therapy and a decreased risk of atrial fibrillation/flutter in patients with and without chronic kidney disease. Atherosclerosis.

[33] Cheng CL, Lee CH, Chen PS, Li YH, Lin SJ, Yang YH (2014). Validation of acute myocardial infarction cases in the national health insurance research database in Taiwan. J. Epidemiol..

[34] Cheng CL, Kao YH, Lin SJ, Lee CH, Lai ML (2011). Validation of the national health insurance research database with ischemic stroke cases in Taiwan. Pharmacoepidemiol. Drug Saf..

[35] Hsing AW, Ioannidis JP (2015). Nationwide population science: lessons from the Taiwan national health insurance research database. JAMA Intern. Med..

[36] Liu CH, Chen TH, Lin MS (2016). Ezetimibe-simvastatin therapy reduce recurrent ischemic stroke risks in type 2 diabetic patients. J. Clin. Endocrinol. Metab..

[37] Li YR, Tsai SS, Lin YS (2017). Moderate- to high-intensity statins for secondary prevention in patients with type 2 diabetes mellitus on dialysis after acute myocardial infarction. Diabetol. Metab. Syndr..

[38] Chung CM, Lin MS, Chang CH (2017). Moderate to high intensity statin in dialysis patients after acute myocardial infarction: A national cohort study in Asia. Atherosclerosis.

[39] Lin CC, Lai MS, Syu CY, Chang SC, Tseng FY (2005). Accuracy of diabetes diagnosis in health insurance claims data in Taiwan. J. Formos Med. Assoc..

[40] Cheng CL, Chien HC, Lee CH, Lin SJ, Yang YH (2015). Validity of in-hospital mortality data among patients with acute myocardial infarction or stroke in National Health Insurance Research Database in Taiwan. Int. J. Cardiol..

[41] Austin PC, Fine JP (2019). Propensity-score matching with competing risks in survival analysis. Stat. Med..

[42] Mendis S, Davis S, Norrving B (2015). Organizational update: the world health organization global status report on noncommunicable diseases 2014; one more landmark step in the combat against stroke and vascular disease. Stroke.

[43] Ference BA, Majeed F, Penumetcha R, Flack JM, Brook RD (2015). Effect of naturally random allocation to lower low-density lipoprotein cholesterol on the risk of coronary heart disease mediated by polymorphisms in NPC1L1, HMGCR, or both: a 2 x 2 factorial Mendelian randomization study. J. Am. Coll. Cardiol..

[44] Libby P, Sasiela W (2006). Plaque stabilization: can we turn theory into evidence?. Am. J. Cardiol..

[45] Nawrocki JW, Weiss SR, Davidson MH (1995). Reduction of LDL cholesterol by 25% to 60% in patients with primary hypercholesterolemia by atorvastatin, a new HMG-CoA reductase inhibitor. Arterioscler. Thromb. Vasc. Biol..

[46] Adams SP, Tsang M, Wright JM (2015). Lipid-lowering efficacy of atorvastatin. Cochrane Database Syst Rev..

[47] Collins R, Reith C, Emberson J (2016). Interpretation of the evidence for the efficacy and safety of statin therapy. Lancet.

[48] Bays HE, Ose L, Fraser N (2004). A multicenter, randomized, double-blind, placebo-controlled, factorial design study to evaluate the lipid-altering efficacy and safety profile of the ezetimibe/simvastatin tablet compared with ezetimibe and simvastatin monotherapy in patients with primary hypercholesterolemia. Clin. Ther..

[49] Catapano AL, Davidson MH, Ballantyne CM (2006). Lipid-altering efficacy of the ezetimibe/simvastatin single tablet versus rosuvastatin in hypercholesterolemic patients. Curr. Med. Res. Opin..

[50] Jarcho JA, Keaney JF (2015). Proof That Lower Is Better–LDL Cholesterol and IMPROVE-IT. N. Engl. J .Med..

[51] Pauwels RA, Rabe KF (2004). Burden and clinical features of chronic obstructive pulmonary disease (COPD). Lancet.

