# Supplementary material for: Similar major cardiovascular outcomes between pure statin and ezetimibe-statin in comparable intensity for type 2 diabetes with extremely atherosclerotic risks
Source: Sci Rep. 2021 Mar 23;11:6697. doi: 10.1038/s41598-021-86090-9 (PMC7988142; doi:10.1038/s41598-021-86090-9)
Supplement: Supplementary file 1 — Supplementary Information 1. [file 41598_2021_86090_MOESM1_ESM.docx]

**Supplemental Table 1**. International Classification of Diseases, Ninth Revision, Clinical Modification codes used for diagnosis in this study

| Variable | ICD-9 CM Code |
| --- | --- |
| Acute coronary syndrome | 410.xx, 411.1, 411.8x |
| Acute ischemic stroke | 433.xx–435.xx, excluded 433.00, 433.10, 433.20, 433.30, 433.80, 433.90, 434.90, 434.00, 434.10, and 434.90 |
| Diabetes mellitus | A180, 250.xx |
| Myocardial infarction | 410.xx, 412.xx |
| Heart failure | 428.xx |
| Venous thromboembolism | 415.1x (pulmonary embolism), 453.xx (deep vein thrombosis) |
| Chronic kidney disease | 580.xx–589.xx, 403.xx–404.xx, 016.0x, 095.4x, 236.9x, 250.4x, 274.1x, 442.1x, 447.3x, 440.1x, 572.4x, 642.1x, 646.2x, 753.1x, 283.11, 403.01, 404.02, 446.21 |
| Dialysis | 585.xx (Catastrophic illness card) |
| Gout | 274.xx |
| Atrial fibrillation | 427.31 |
| Peripheral arterial disease | 440.0x, 440.2x, 440.3x, 440.8x, 440.9x, 443.xx, 444.0x, 444.22, 444.8x, 447.8x, 447.9x |
| Hypertension | 401.xx–405.xx |
| Dyslipidemia | 272.xx |
| Chronic obstructive pulmonary disease | 491.xx, 492.xx, 496.xx |
| Malignancy | 140.xx–208.xx (Catastrophic illness card) |
| Cirrhosis | 571.5, 571.6, 571.2 |
| Hepatitis B virus infection | 070.20, 070.22, 070.30, 070.32, and V02.61 |
| Hepatitis C virus infection | 070.41, 070.44, 070.51, 070.54, 070.70, 070.71, V02.62 |
| Alcoholism | V113, 291.xx, 305.0x, 357.5, 425.5, 303.xx, 571.0, 571.1, 571.2, 571.3, 980.0 |
| Autoimmune disease | 710.0, 710.1, 714.0, 710.4, 710.3, 446.0, 446.2, 446.4, 446.5, 443.1, 446.7, 136.1, 694.4, 710.2, 555.xx, 556.xx |
| Cardiovascular death | 390.xx–459.xx, 785.5x |
| Stroke | 430.xx–437.xx |
| Unstable angina | 411.1, 411.8x |
| Hemorrhagic stroke | 430.xx–432.xx |
| Dementia | 290.xx, 294.xx |
| Rhabdomyolysis | 728.88, 728.89 |
| Acute hepatitis | 277.4, 570, 572.8, 573.3, 573.8, 576.8, 782.4 |

ICD-9 CM, International Classification of Diseases, Ninth Revision, Clinical Modification.
