# Supplementary material for: Similar major cardiovascular outcomes between pure statin and ezetimibe-statin in comparable intensity for type 2 diabetes with extremely atherosclerotic risks
Source: Sci Rep. 2021 Mar 23;11:6697. doi: 10.1038/s41598-021-86090-9 (PMC7988142; doi:10.1038/s41598-021-86090-9)
Supplement: Supplementary file 2 — Supplementary Information 2. [file 41598_2021_86090_MOESM2_ESM.docx]

**Supplemental Table 2.** Medication possession ratio

|  | Before matching | | |  | After matching | | |
| --- | --- | --- | --- | --- | --- | --- | --- |
| Variable | ATOR 40  (*n* = 5,249) | EZ-SIM 20  (*n* = 1,710) | *P* |  | ATOR 40  (*n* = 1,686) | EZ-SIM 20  (*n* = 1,686) | *P* |
| MPR (%) | 47.9 ± 40.0 | 53.3 ± 40.2 | <0.001 |  | 52.6 ± 42.7 | 53.0 ± 40.2 | 0.777 |

ATOR 40, atorvastatin 40mg; EZ-SIM 20, ezetimibe 10mg/simvastatin 20mg; MPR, medication possession ratio

Data presented as mean ± standard deviation
