# Supplementary figures and images for: Similar major cardiovascular outcomes between pure statin and ezetimibe-statin in comparable intensity for type 2 diabetes with extremely atherosclerotic risks
Source: Sci Rep. 2021 Mar 23;11:6697. doi: 10.1038/s41598-021-86090-9 (PMC7988142; doi:10.1038/s41598-021-86090-9)

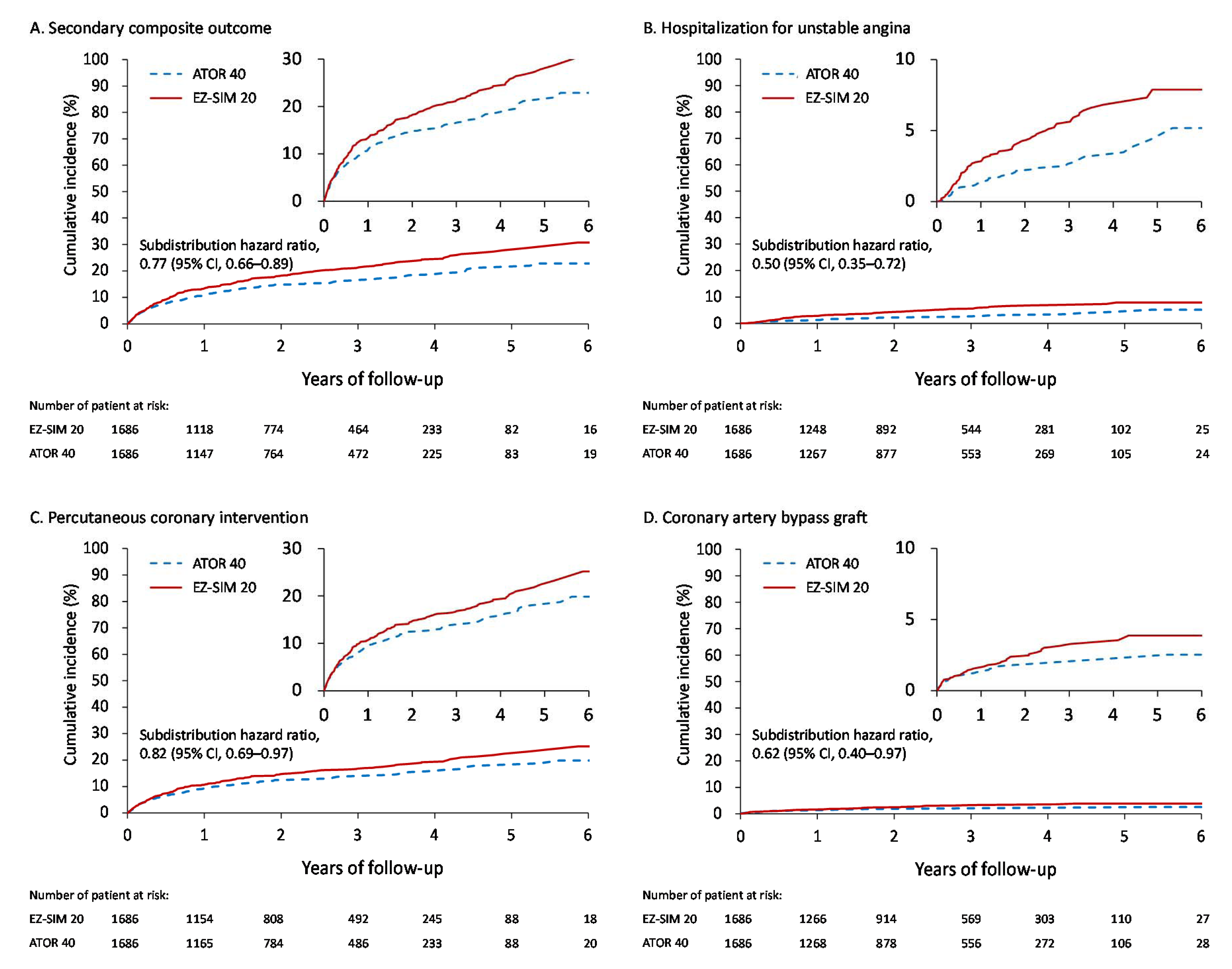

Supplement: Supplementary file 3 — Supplementary Information 3. [file 41598_2021_86090_MOESM3_ESM.tif]

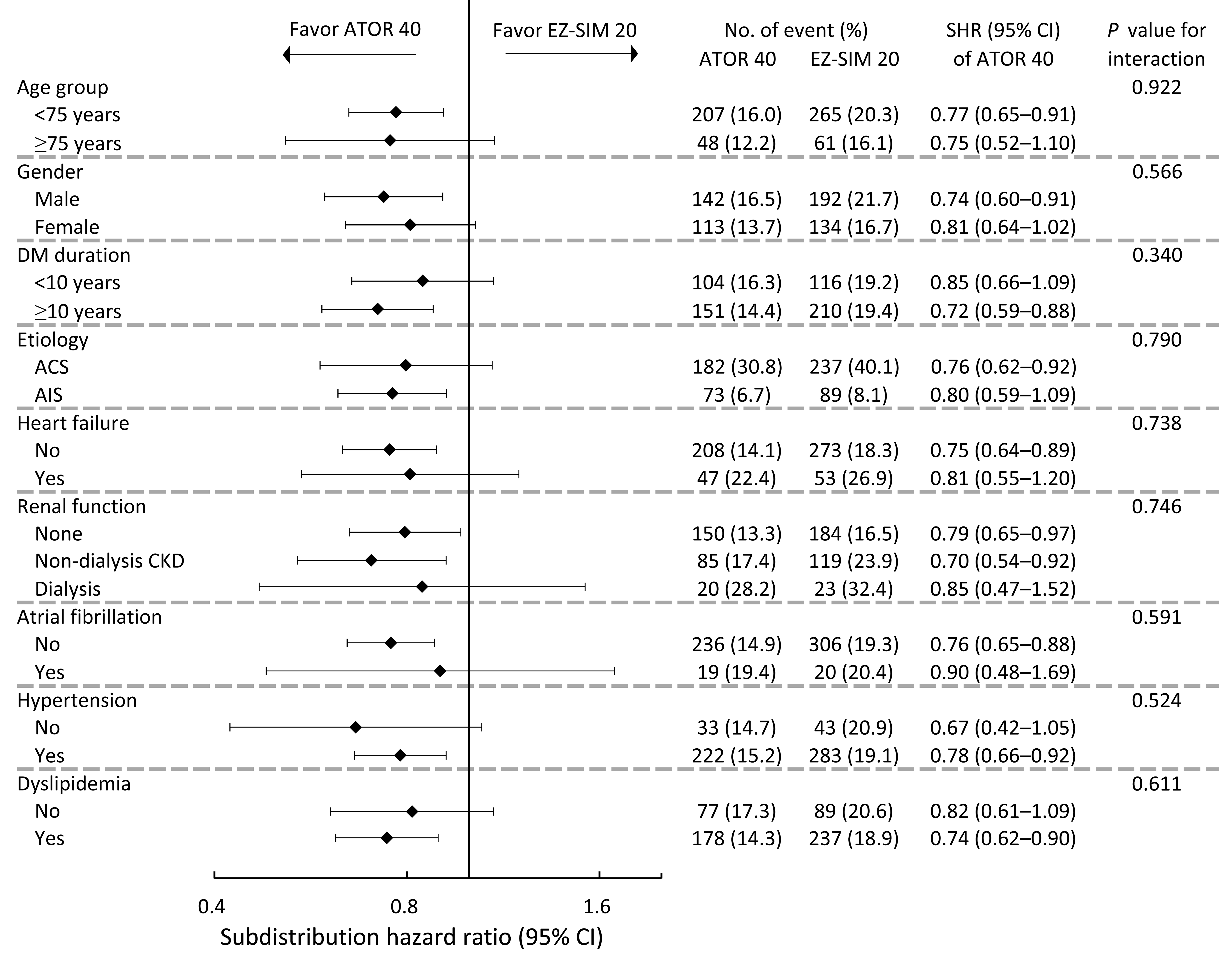

Supplement: Supplementary file 4 — Supplementary Information 4. [file 41598_2021_86090_MOESM4_ESM.tif]
