# Supplementary material for: Similar major cardiovascular outcomes between pure statin and ezetimibe-statin in comparable intensity for type 2 diabetes with extremely atherosclerotic risks
Source: Sci Rep. 2021 Mar 23;11:6697. doi: 10.1038/s41598-021-86090-9 (PMC7988142; doi:10.1038/s41598-021-86090-9)
Supplement: Supplementary file 5 — Supplementary Legends. [file 41598_2021_86090_MOESM5_ESM.docx]

**Legend for supplemental table and figure**

**Supplemental Table 1.** International Classification of Diseases, Ninth Revision, Clinical Modification codes used for diagnosis in this study

**Supplemental Table 2.** Medication possession ratio

**Supplemental Figure 1.** The cumulative incidence of the secondary composite outcome (A), and the individual outcomes of hospitalization for unstable angina (B), percutaneous coronary intervention (C) and coronary artery bypass grafting (D) in the propensity score matched cohorts

**Supplemental Figure 2.** The *post-hoc* subgroup analysis of the secondary composite outcome
